# An Epidemiologic Investigation of Potential Risk Factors for Nodding Syndrome in Kitgum District, Uganda

**DOI:** 10.1371/journal.pone.0066419

**Published:** 2013-06-18

**Authors:** Jennifer L. Foltz, Issa Makumbi, James J. Sejvar, Mugagga Malimbo, Richard Ndyomugyenyi, Anne Deborah Atai-Omoruto, Lorraine N. Alexander, Betty Abang, Paul Melstrom, Angelina M. Kakooza, Dennis Olara, Robert G. Downing, Thomas B. Nutman, Scott F. Dowell, D. K. W. Lwamafa

**Affiliations:** 1 Division of Nutrition, Physical Activity and Obesity, Centers for Disease Control and Prevention, Atlanta, Georgia, United States of America; 2 Uganda Ministry of Health, Kampala, Uganda; 3 Division of High-Consequence Pathogens and Pathology, Centers for Disease Control and Prevention, Atlanta, Georgia, United States of America; 4 Division of Global HIV/AIDS, Centers for Disease Control and Prevention Uganda, Entebbe, Uganda; 5 Division of Environmental Hazards and Health Effects, Centers for Disease Control and Prevention, Atlanta, Georgia, United States of America; 6 School of Medicine, Makerere University College of Health Sciences, Kampala, Uganda; 7 Laboratory of Parasitic Diseases, National Institutes of Health, Bethesda, Maryland, United States of America; 8 Division of Global Disease Detection and Emergency Response, Centers for Disease Control and Prevention, Atlanta, Georgia, United States of America; Institute of Neuroepidemiology and Tropical Neurology, France

## Abstract

**Introduction:**

Nodding Syndrome (NS), an unexplained illness characterized by spells of head bobbing, has been reported in Sudan and Tanzania, perhaps as early as 1962. Hypothesized causes include sorghum consumption, measles, and onchocerciasis infection. In 2009, a couple thousand cases were reportedly in Northern Uganda.

**Methods:**

In December 2009, we identified cases in Kitgum District. The case definition included persons who were previously developmentally normal who had nodding. Cases, further defined as 5- to 15-years-old with an additional neurological deficit, were matched to village controls to assess risk factors and test biological specimens. Logistic regression models were used to evaluate associations.

**Results:**

Surveillance identified 224 cases; most (95%) were 5–15-years-old (range = 2–27). Cases were reported in Uganda since 1997. The overall prevalence was 12 cases per 1,000 (range by parish = 0·6–46). The case-control investigation (n = 49 case/village control pairs) showed no association between NS and previously reported measles; sorghum was consumed by most subjects. Positive onchocerciasis serology [age-adjusted odds ratio (AOR_1_) = 14·4 (2·7, 78·3)], exposure to munitions [AOR_1_ = 13·9 (1·4, 135·3)], and consumption of crushed roots [AOR_1_ = 5·4 (1·3, 22·1)] were more likely in cases. Vitamin B6 deficiency was present in the majority of cases (84%) and controls (75%).

**Conclusion:**

NS appears to be increasing in Uganda since 2000 with 2009 parish prevalence as high as 46 cases per 1,000 5- to 15-year old children. Our results found no supporting evidence for many proposed NS risk factors, revealed association with onchocerciasis, which for the first time was examined with serologic testing, and raised nutritional deficiencies and toxic exposures as possible etiologies.

## Introduction

An unusual syndrome referred to as “nodding disease” or “nodding syndrome” (NS) due to characteristic spells of repetitive head bobbing has been reported among populations in Africa, perhaps as early as 1962 in Tanzania. [1] The syndrome is characterized by stereotypic head nodding, along with variable other features including apparent seizures, cognitive and physical impairment, and progressive functional difficulties, with onset during childhood. In the late 1900s, “rhythmic dorsoventral head movements” in Liberia [2], [3] and “repetitive head movements” in Western Uganda [4] were noted to resemble the same “head nodding” though they may not represent one distinct seizure type. More recently, the syndrome termed NS has been investigated among internally displaced people in southern Sudan in 2001–02 [5], [6] and among children in the Mahenge region in Tanzania in 2005. [7], [8].

The underlying cause, natural history and pathophysiology remain unclear despite prior investigations. The primary feature of NS is a paroxysmal “spell” in which the head bobs forward repeatedly over a period of minutes; nodding is often reported to happen at the sight of food, with cold temperatures, and “when the moon appears.” In most cases the child appears unresponsive during the episode. Children with NS have variably been described to develop other physical and neurological signs, primarily growth stunting, and cognitive regression. [5], [8] The nodding episodes are thought to be the sentinel feature of a more progressive neurological illness, in which there is neurological deterioration including progressive seizures. [5], [8] Electroencephalography (EEG) results from a prior study suggest that at least a subset of children with head nodding have other types of clinical and subclinical seizures. [7] Studies, including brain magnetic resonance imaging and cerebrospinal fluid (CSF) analysis, have not suggested an etiology. [7] Anecdotal reports have described the natural history of the illness as one of progressive neurologic deterioration, physical wasting, and ultimately death. In focus groups, Ugandans report symptoms consistent with the NS described in other sub-Saharan countries (UgMOH, WHO, CDC, unpublished report).

Proposed risk factors or causes have included psychogenic illness, infectious agents, genetic disease, toxins, and nutritional disorders. Exposures that were found to have significant associations with NS in Sudan included onchocerciasis, [9] a self-reported history of measles, and red sorghum grain consumption (Anker, unpublished report).

In 2009, the Kitgum District Health Office reported to the Uganda Ministry of Health approximately 2,000 cases suggestive of NS among persons previously displaced by war. Given the complex nature of the disease and the range of possible etiologies, the U.S. Centers for Disease Control and Prevention was asked to assist the Ministry to investigate and respond to the outbreak. We conducted a case-control investigation to identify potential risk factors and etiologies for development of NS, and to direct control efforts.

## Methods

In December 2009, we conducted a multidisciplinary investigation including surveillance, case-control, clinical case-series, and qualitative assessments in western Kitgum District, Uganda. The clinical case-series as well as the focus groups which informed questionnaire development and interpretation of results are described elsewhere. [10] For surveillance purposes, we defined a case of NS as a previously developmentally normal person of any age with nodding episodes where the head drops forward repeatedly (Table 1). To narrow the definition for the case-control investigation, a case also was required to have another neurological abnormality. Age was limited to 5- to 15-years-old for cases and controls, the age group in which most children with the syndrome fell.

**Table 1:** Case definitions for persons with Nodding Syndrome. Criteria 1–2 were required^∧^ for surveillance purposes and 1–5* were required for enrollment in the case-control investigation.

1	The presence of one or more episodes of head nodding. Head nodding was defined as repetitive dropping of the head with periodicity of 10–30 times over a period of 1 minute or more.
2	A child who was developmentally normal before onset. Developmentally normal was defined as meeting developmental milestones by parental report.
3	Age 5 to 15 years
4	At least one other definitive neurological abnormality, which could be either reported or observed. Definitive neurological abnormalities included loss of developmental milestones, generalized tonic-clonic seizures, objective neurological exam findings (e.g. spasticity, ataxia), or other clear sign of neurological deficit.
5	Lack of improvement

∧ as determined by Uganda Ministry of Health and WHO epidemiologists.

* as determined by CDC medical epidemiologist.

Cases were recruited across western Kitgum District through surveillance that included radio announcements and community outreach by community leaders and health workers to affected families and targeted NS populations. Potential cases were interviewed in-person by surveillance teams using a structured questionnaire. Only subjects meeting the surveillance case definition were counted.

For the concurrent case-control investigation, cases (total n = 51) were matched on location to an unrelated village control who was not living in the same household as the case (n = 49) and/or to a sibling in the household (n = 44) who were neurologically normal. Based upon the results of previous investigations, focus group interviews of community members (UgMOH, WHO, CDC, unpublished report), and the input of subject matter experts, we generated a list of possible risk factors including infectious diseases, nutritional deficiencies, heavy metals, pesticides, war munitions, post-traumatic stress, mass hysteria, pseudoseizures, and genetic disorders.

After informed consent, in-person interviews were conducted in the native Acholi language through translators with a health background, by physicians, other health providers, and non-clinical researchers, to collect exposure information and clinical findings. Respondents were also asked: where they were living when the nodding began to assess if location at symptom onset contributed to NS risk; whether a family member had been abducted to assess for potential social stressors; and about occurrence of visual or auditory hallucinations and school attendance to explore potential disease manifestations in cases. Additionally, on physical examination we assessed for skin nodules, a feature characteristic of onchocerciasis. Measurement of height and weight were obtained using a stadiometer and digital scale to calculate anthropometric indices including body mass index (BMI, kg/m^2^), low BMI-for-age z-score (defined as <−2 standard deviations (SD)), and low height-for-age z-score (<−2SD).

Laboratory specimens collected on all subjects included serum, whole blood, and urine. Two skin snips were taken from the iliac crests of all subjects with a 2 mm Holth corneoscleral punch and microscopically examined after incubation for 30 minutes in distilled water (and a further 24 hours in saline for negative skin snips) for the presence and number of *O. volvulus* microfilariae. [11] When selecting a subset of cases for CSF testing, we prioritized cases with a high clinical likelihood of head nodding. Cell counts were not performed on freshly obtained CSF due to the remote field location. Specimens were appropriated to various laboratories for testing: skin snip *O. volvulus* microfilariae (counted in the field by Uganda MOH parasitologists); ALT, AST, alkaline phosphatase, creatinine, urea, bilirubin (St. Joseph’s Hospital, Kitgum, Uganda); *O. volvulus* Ov16, OvFAR/MSA serology (Laboratory of Parasitic Diseases, U.S. National Institutes of Health); Hepatitis E serology (Uganda Virus Research Institute); *Taenia solium* serology (CDC Parasitic Diseases Branch); *Trypanosoma gambiense* serology (Prince Leopold Institute for Tropical Medicine Parasite Diagnostics Unit); RBC folate, serum vitamins B6, B12, A (CDC Nutritional Biomarkers Branch); serum zinc, selenium, copper, urine mercury (CDC Inorganic and Radiation Analytical Toxicology Branch); urine homocysteine (ARUP commercial national reference laboratory); urine thiocyanate (CDC Emergency Response Branch), serum and CSF multiplex PCR (CDC Gastroenteritis and Respiratory Virus Laboratory Branch); CSF protein, glucose (Children’s Hospital of Atlanta Cytology Laboratory); and CSF measles PCR (CDC Measles, Mumps, Rubella and Herpes Virus Laboratory Branch). We focused on testing for exposures thought to cause CNS disease, hypothesized to be associated with NS previously, or recognized to have caused epidemics in the region during the years preceding the rise in NS cases. Tests were run on all available specimens when possible. Because of resource limitations, some laboratory tests (vitamin B12, vitamin A, zinc, selenium, copper, folate, mercury, homocysteine) were run on a subsample of available specimens selected by using a random digit table.

### Analysis

Prevalence was calculated for each parish visited by dividing the number of 5- to 15-year-old cases by the number of 5- to 15-year-olds in each subcounty using 2009 census data. Conditional logistic regression models were used to estimate the associations between NS and exposures or clinical findings; unadjusted and adjusted odds ratios and 95% confidence intervals (CI) were calculated. Because cases were significantly older than controls in our sample, all final models were age-adjusted. We examined the association between each individual exposure and disease, using three strategies to control for potential covariates. The first adjusted for age (model 1); the second adjusted for significant exposures in our investigation including munitions, crushed roots, age (model 2); and the third adjusted significant variables from previous studies including history of prior measles, sorghum consumption, onchocerciasis skin snip positive, plus age (model 3). We tested for all two-way interactions between munitions, crushed roots, and age as well as all two-way interactions between measles, sorghum, onchocerciasis skin snips, and age; Wald chi-square tests were used to assess the significance of interaction terms. Given the smaller sample size for most laboratory results, unmatched multiple logistic regression was used to explore unadjusted and age-adjusted associations between lab results and case status. SAS software (version 9·2; SAS Institute) was used for analyses.

### Human Subjects

This investigation was reviewed in accordance with CDC human subjects review procedures and determined to be a non-research public health response activity by not meeting the regulatory definition of research under 45 CFR 46.102(d), with concurrence from the Uganda Ministry of Health. The purpose and the nature of the investigation were explained to parents/caregivers, including risks and benefits of each of the procedures and evaluations; all parents/caregivers provided written informed consent for their children to be included in the investigation. Parents/caregivers were free to decline participation. Measures were maintained to protect patient confidentiality.

## Results

**Figure 1:**
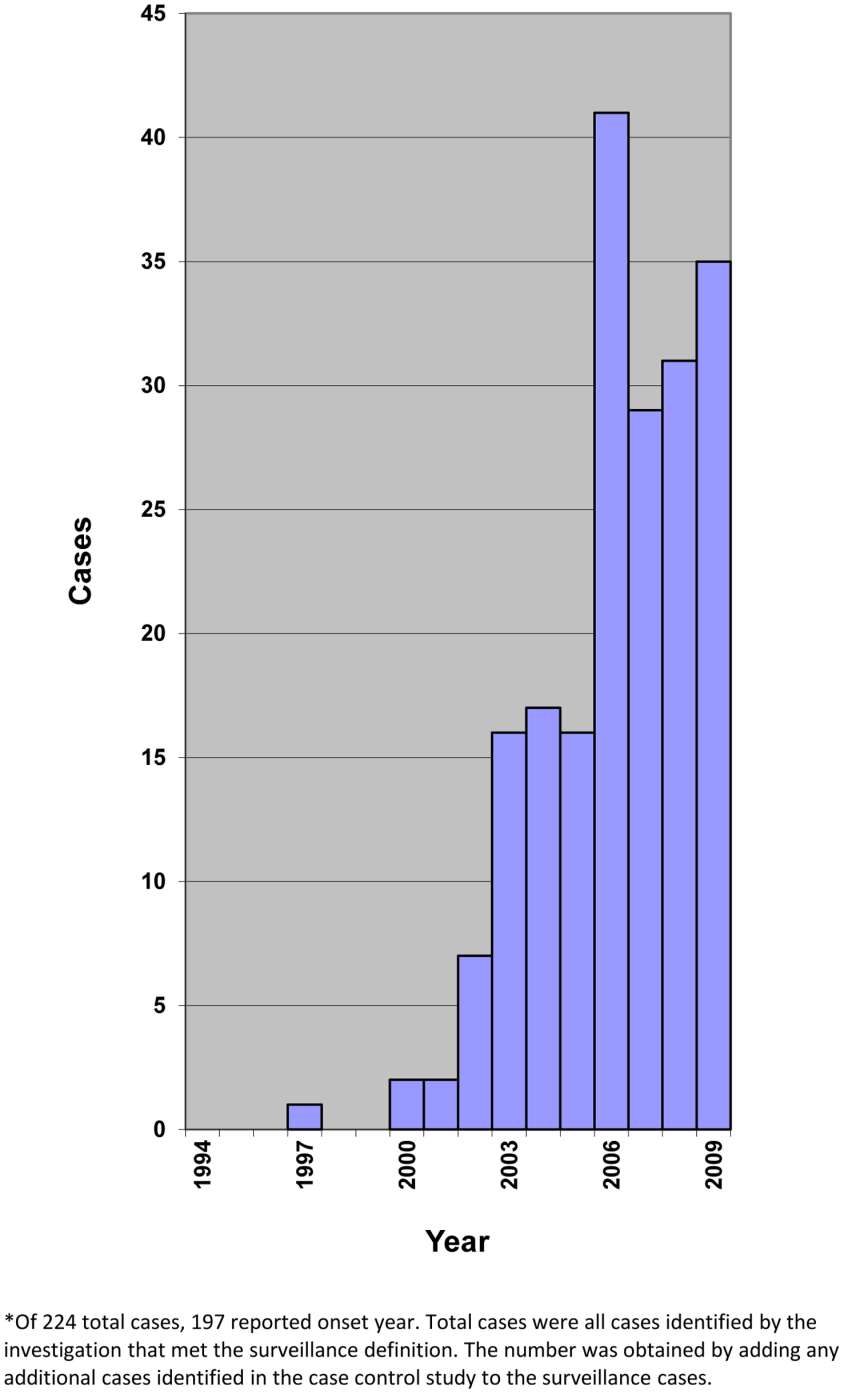
Epidemic curve depicting time course of disease onset: cases identified by surveillence and case-control studies in Kitgum District plotted by reported NS onset year.*.

We identified 224 persons who met the surveillance case definition (n = 30 cases unique to the case-control study, n = 173 unique to surveillance, n = 21 in both studies), of whom 95% were between the ages of 5 and 15 years (range = 2–27) (Figure S1; available online). Except for a single case with onset reported in 1997, all cases in this northern Ugandan population reported onset after 2000, and there was a generally higher number of reported cases annually through 2009 (Figure 1). Analysis of disease onset by month revealed no evidence of seasonal variation. The overall prevalence in 13 parishes of western Kitgum District was 12 (exact 95% CI = 10·8, 14·2) cases per 1000 five- to fifteen-year-old children, but ranged widely from 0·6 (0, 3·6) to 46 (36·5, 57·7) cases per 1000.

All case-control subjects were formerly internally displaced people (i.e. previously living in another area of Uganda due to war) now living in Kitgum district (Table 2). Despite testing controls within the same age range as cases, cases were significantly older than controls and later in birth order. All other socio-demographic characteristics were similar between cases and controls. Most subjects were cared for by parent(s) who had less than a primary school education and were unemployed, and had a family member who had been abducted.

**Table 2:** Prevalence and mean socio-demographic characteristics of nodding syndrome case patients and control subjects aged 5 to 15 years in Kitgum District, Uganda.

Characteristic	Cases	Village Controls	Household Controls
*Total subjects*	51∧	49	44
*Male gender*	55%	44%	45%
*Age*			
Mean	11·6 years	8·5 years**	8·1 years**
*Birth Order*			
Mean	4·1	3·2*	4·9**
Range	1–9	1–10	1–10
*Parent caretaker*	86%	84%	86%
*Family member abducted*	59%	49%	57%
*Father’s education*			
None	10%	17%	8%
<Primary school	57%	46%	55%
Graduated primary school or above	33%	37%	37%
*Mother’s education*			
None	34%	43%	42%
<Primary school	58%	45%	51%
Graduated primary school or above	8%	13%	7%
*Primary caretaker is employed*	6%	6%	5%

∧ 51 total cases were enrolled; 49 matched to 49 village controls, and 44 matched to 44 household controls.

McNemar’s, Stuart’s [27], and paired t tests were performed to obtain significance level.

* p<0.05.

** p<0.001.

Cases were more likely to report exposure to munitions prior to disease onset than controls queried for the same time period (Table 3). Cases also had higher age-adjusted odds of crushed root consumption. Findings indicated no difference in age-adjusted odds of sorghum consumption or self-reported history of measles. Results remained similar with adjusted models 2 and 3 (Table S1; available online) and no significant interactions were found. For location at the time of NS onset in the matched case, most cases and controls were living in Kitgum Town Council (39% and 31%, respectively), Namokora/Atiak (35%, 29%), or Palabek Gem (20%, 25%) subcounties. For anthropometric indices, 60% of cases had low height-for-age as compared to 29% of controls (p = 0·003). Cases also had higher frequencies of low BMI-for-age (42%) as compared to controls (13%, p = 0·001). In addition to the neurological signs included in the case definition, cases were also more likely to report visual hallucinations and to have dropped out of school. (Of note, further investigation with culturally appropriate clinical assessments should be made for any clinical findings as case-control questioning is appropriate for uncovering reported differences from a control population but should not serve as the primary information on which to base these clinical care decisions.) Additional clinical findings from the case-series investigation, including details on the gross cognitive impairment in cases and atonic seizures causing nodding, are presented separately. [10].

**Table 3:** Frequency of nodding syndrome cases and village controls with positive exposures or presence of clinical findings.

	Positive Cases %*		Positive Controls %			OR‡ (95%CI)		AOR Model 1† (95%CI)	
**Exposure**									
Ever treated for Onchocerciasis	33·3		24·5			1·7 (0·6, 4·6)		1·1 (0·3, 3·8)	
Family member abducted	58·8		49·0			1·5 (0·6, 3·7)		1·0 (0·3, 2·9)	
*History of*									
Measles	23·5		6·1			**4·0 (1·1, 14·2)**		3·3 (0·8, 13·6)	
Malaria^∧^	43·1		59·2			0·5 (0·2, 1·2)		0·7 (0·2, 1·9)	
Malnutrition^∧^	3·9		4·1			0·6 (0·1, 2·7)		1·0 (0·2, 4·8)	
Pneumonia^∧^	0·0		2·0			0·4 (0·0, 16·5)+		0·6 (0·0, 35·1)+	
Diarrhea^∧^	2·0		4·1			0·7 (0·2, 3·4)		0·7 (0·1, 3·7)	
Head injury^∧^	2·0		0·0			0·4 (0·0, 3·3)+		0·2 (0·0, 1·8)+	
Tapeworm	0·0		0·0			–		–	
*Consumption of*									
Red sorghum	98·0		100			0·2 (0·0, 15·4)+		1·3 (0·0, 125·9)+	
Spoiled relief foods	43·1		46·9			0·7 (0·2, 2·3)		0·3 (0·1, 1·3)	
Supplementary foods	21·6		12·2			1·8 (0·6, 5·4)		1·5 (0·4, 5·5)	
Seeds meant for planting	60·8		65·3			0·9 (0·3, 2·6)		0·6 (0·1, 2·3)	
River Fish	96·1		100			0·3 (0·0, 11·6)+		0·3 (0·0, 11·6)+	
Insects	41·2		32·7			1·5 (0·5, 4·2)		0·8 (0·2, 2·9)	
Rodent brain	54·9		51·0			2·0 (0·4, 10·9)		1·8 (0·3, 12·3)	
Guinea fowl brain	7·8		4·1			2·5 (0·5, 12·9)		2·4 (0·4, 14·8)	
Bush meat	100		100			–		–	
Cassava	100		100			–		–	
*Use of traditional or herbal medicines*									
Crushed roots^∧^		39·2		16·3	**5·0 (1·4, 17·3)**		**5·4 (1·3, 22·1)**		
Crushed leaves^∧^		7·8		2·0	4·0 (0·4, 35·8)		3·4 (0·2, 45·8)		
Crushed flowers^∧^		0·0		2·0	0·7 (0·1, 4·3)		0·9 (0·1, 5·6)		
Inhaled medicine^∧^		2·0		0·0	0·3 (0·0, 3·0)+		0·2 (0·0, 1·5)+		
Lotion		0·0		0·0	–		–		
Broth		0·0		0·0	–		–		
*Water source for domestic use*									
Rivers/streams		80·7		83·7	0·1 (0·0, 5·2)+		0·1 (0·0, 3·6)+		
Boreholes		96·1		100	0·1 (0·0, 4·1)+		0·3 (0·0, 14·5)+		
Shallow wells		11·8		6·1	4·0 (0·4, 35·8)		3·4 (0·2, 45·8)		
Springs		5·9		4·1	1·5 (0·3, 9·0)		2·6 (0·3, 21·4)		
Piped water		0·0		0·0	–		–		
*Exposure to*									
Munitions		70·6		51·0	**10·0 (1·3, 78·1)**		**13·9 (1·4, 135·3)**		
Unusual illness/death of animals		52·9		53·1	0·6 (0·2, 1·9)		0·6 (0·1, 2·3)		
Swimming in the river^∧^		17·7		22·5	0·7 (0·2, 2·4)		0·3 (0·1, 1·7)		
Swimming in the pond^∧^		7·8		4·1	3·0 (0·3, 28·8)		1·9 (0·1, 36·0)		
**Clinical Finding**									
*Physical examination*									
Skin Nodules^∧^		7·8		8·2	1·0 (0·3, 4·0)		0·5 (0·1, 2·6)		
Low height for age^∧^, **		60·0		29·2	**3·6 (1·3, 9·7)**		2·5 (0·8, 7·7)		
Low BMI for age^∧^, **		42·2		12·5	**4·0 (1·3, 12·0)**		2·0 (0·5, 7·4)		
*Clinical characteristic*									
Visual Hallucinations^∧^		29·4		4·1	**14·0 (1·8, 106·5)**		**13·6 (1·5, 121·7)**		
Auditory Hallucinations^∧^		25·5		0	**44·9 (2·3, 858·3)** +		14·9 (0·7, 299·1)+		
School Attendance		52·9		85·7	**0·2 (0·1, 0·6)**		**0·1 (0·0, 0·6)**		

Statistically significant values are in bold.

CI: Confidence interval. OR: odds ratio.

* Percent with exposure is calculated by number of cases with a positive exposure divided by number of cases, or number of controls exposed divided by number of controls.

‡ Odds ratio calculated as odds of positive exposure in cases versus odds of exposure in controls.

† AOR_1_: Odds ratio adjusted for age. Note: additional models 2 (adjusted for age, munitions, roots) and 3 (adjusted for age, measles, sorghum, onchocerciasis skin snip positive) are available in an online appendix Table 5.

? Missing data existed for the following exposure variables: malaria, malnutrition, pneumonia, diarrhea, head injury, crushed leaves, roots, flowers, inhaled medicine (number of cases responding to question = 50); swimming in the river or pond, visual or auditory hallucinations (cases = 49); skin nodules (cases = 48); low height for age, low BMI for age (cases = 45, controls = 48); all data used for frequencies, data from available matched pairs used for matched analyses.

+ Firth’s correction.

** low BMI-for-age z-score: <−2 SD, an indicator of acute malnutrition; low height-for-age z-score: <−2SD, chronic malnutrition.

Unadjusted and adjusted odds of positive exposure or clinical finding in a case versus control.

Laboratory results demonstrated no significant difference between cases and controls for most variables (Table 4). However, cases were more likely than controls to have *O. volvulus*-specific IgG antibodies by both serologic assays. Also, most cases and controls had low vitamin B6 levels, as measured by serum pyridoxal-5′-phosphate (PLP) [12]. We explored whether the co-occurrence of the two exposures, positive onchocerciasis and low serum PLP, were associated with case status, however there was no evidence of a significant interaction between the two.

**Table 4:** Comparison of laboratory findings among nodding syndrome cases and village controls, frequency for positive results or high value unless otherwise denoted by “*”, unmatched unadjusted and age-adjusted odds of an abnormal laboratory value∧, †, ¶ in a case.

	Cases		Controls		OR	AOR**
	n	%	n	%	(95%CI)	(95%CI)
*Skin snip*						
* Onchocerca volvulus* microfilariae†††	45	71·1	39	53·9	2·11 (0·86, 5·2)	1·11 (0·37, 3·27)
*Serum*						
* O· volvulus* antibody Ov16 by ELISA¶¶¶	39	66·7	44	31·8	**4·29 (1·71, 10·75)**	**3·14 (1·08, 9·13)**
* O· volvulus* antibodies OvFAR/MSA by LIPS∧∧ ¶¶¶	39	94·9	41	48·8	**19·42 (4·13, 91·43)**	**14·40 (2·65, 78·31)**
Hepatitis E IgM	38	31·6	31	16·1	2·31 (0·71, 7·50)	1·45 (0·37, 5·68)
Hepatitis E IgG	38	26·3	30	33·3	0·71 (0·25, 2·04)	0·81 (0·24, 2·75)
Hepatitis E IgM/G	38	47·4	31	41·9	1·18 (0·45, 3·08)	1·03 (0·33, 3·21)
* Taenia soleum* (cystercercosis) antibody	36	0	40	0	–	–
* Trypanosoma gambiense* antibody by CATT††	36	0	40	0	–	–
Pyridoxal-5′-phosphate (vitamin B6)* by HPLC¶¶fluorometric detection	41	73·2	42	64·3	1·52 (0·59, 3·86)	1·22 (0·41, 3·59)
Cobalamin (vitamin B12)* by immunoassay	25	8·0	12	8·3	0·96 (0·08, 11·72)	1·46 (0·09, 22·82)
Retinol (vitamin A)* by HPLC-UV/VIS***	25	40·0	12	33·3	1·33 (0·32, 5·64)	2·15 (0·41, 11·12)
Zinc*	17	47·1	12	66·7	0·44 (0·10, 2·06)	0·72 (0·13, 3·94)
Selenium*	17	100	12	100	–	–
Copper*	17	0	12	0	–	–
Alanine transaminase (ALT)	44	11·4	42	7·1%	1·67 (0·37, 7·46)	1·96 (0·34, 11·40)
Aspartate aminotransferase (AST)	44	59·1	42	64·3	0·80 (0·34, 1·92)	1·23 (0·41, 3·67)
Alkaline phosphatase	44	22·7	42	14·3%	1·77 (0·58, 5·38)	1·28 (0·35, 4·67)
Creatinine*	44	75·0	42	71·4%	1·20 (0·46, 3·12)	2·76 (0·83, 9·20)
Urea*	44	15·9	42	4·8	3·78 (0·74, 19·39)	3·70 (0·60, 22·86)
Total Bilirubin	44	0	42	0	–	–
Multiplex PCR∧∧∧	42	0	0	–	–	–
*Whole blood*						
Malaria thin and thick smears	22	2·3	21	4·7	0·45 (0·04, 5·40)	0·35 (0·01, 11·72)
Hemoglobin*	43	48·8	39	69·2	0·42 (0·17, 1·05)	0·76 (0·25, 2·31)
RBC Folate* by microbiological assay	11	9·1	9	0	–	–
*Urine*						
Mercury	12	0	10	0	–	–
Thiocyanate	41	7·3	43	7·0	–	–
Homocysteine by spectrophotometry	23	0	9	0	–	–
*Cerebrospinal fluid*						
Protein	14	0	0	–	–	–
Glucose	14	7·1	0	–	–	–
Measles PCR	16	0	0	–	–	–
Multiplex PCR∧∧∧	16	0	0	–	–	–

* indicates values that where “low” or below normal cut off values and signifies an abnormal test result.

∧ Cut off values for positive infectious disease results: onchocerciasis skin snip>0, onchocerciasis Ov16 ELISA >0·1, onchocerciasis OvFAR/−MSA LIPS >7000 (negative<5000) cysticercosis >0, trypanosomiasis >0, hepatitis E >0·22 (Hep E IgM/G was considered reactive if either IgM or IgG was >0·22), measles PCR >0.

† Low values for nutrition results: vitamin B6<20 nmol/L, B12<200 pg/mL, A <20 µg/dL, Hb 12 g/dL, folate <317 nmol/L.

¶ Normal reference ranges: ALT 5·3–43·3 U/l, AST 0–37 U/l, ALP 98–279 U/L, CREA 0·5–1·2 mg/dl, UREA 10–50 mg/dl, TBIL 0–1 mg/dl, copper 60–249 µg/dL, selenium 113–130 µg/L, zinc 70–120 µg/dL, mercury 0·42–3·19 µg/L, thiocyanate <2100 ng/ml (zero cases and controls had toxic levels >14000 ng/ml); CSF protein 15–45 mg/dL; CSF glucose 40–70 gm/dL.

** AOR: Odds ratio adjusted for age.

∧∧ LIPS – *Luciferase immunoprecipitation system.*

†† CATT – Card Agglutination Test with Stained Trypanosomes.

¶¶ HPLC – High-performance liquid chromatography.

*** HPLC-UV/VIS – *HPLC method with ultraviolet-visible spectrophotometry detection.*

∧∧∧ Multiplex PCR tests for 35 groups of family, subfamily or genus nucleic acid targets, covering 19 families of viruses.

††† Case mf density mean = 39·1 (range 0–204); control mf density = 5·8 (range 0–60).

¶¶¶ Matched unadjusted and age-adjusted odds were also performed on labs found to have significant associations: O. volvulus Ov16 OR = 3.33 (1.34, 8.30) and AOR = 3.16 (0.84, 11.89), O. volvulus OvFAR/−MSA OR = 17.00 (2.26, 127.74) and AOR = 20.80 (1.37, 316.24).

Results of analyses comparing cases to household controls or to all controls found no additional associations.

## Discussion

We provide evidence for a recent epidemic of NS in Kitgum district localized geographically to northern Uganda. Similar to reports from Tanzania [7] and Sudan [13], the age distribution of the affected population is clustered tightly among the 5–15 year old age group. Our findings on risk factors provide additional data supporting the association of onchocerciasis with NS, but did not support other previous hypotheses. Additionally, new associations emerged including exposure to munitions and consumption of crushed roots; and both cases and controls were found to have an unusual micronutrient profile.

We found the number of children with reported NS onset to be higher in more recent years. This suggests that the number of NS cases may be increasing in Kitgum district since 2002. Alternate reasons, such as moving away or death, could potentially explain why onset was less frequent in earlier years though this was not substantiated in qualitative interviews done as part of this investigation.

We identified munitions and crushed roots as significant risk factors that have not been assessed in prior investigations. We hypothesized that munitions exposure might indicate potential chemical exposure. However, no known exposures of this type were revealed in focus groups countries (UgMOH, WHO, CDC, unpublished report) or in open-ended questionnaire responses of this case-control study. Instead some respondents indicated the exposure was to guns during raids. Since the munitions association also had a wide confidence interval, the importance of this finding is unclear and warrants further investigation.

Crushed roots were consumed as traditional medicines at higher rates among cases than controls. We were concerned that cassava roots might have caused neurotoxicity but testing for urinary thiocyanates, one indicator of cyanide toxicity from cassava consumption, provided no evidence for ongoing toxicity. Although the roots may have contributed to the development of NS, it is also possible that some respondents confused the question specifying exposures prior to NS onset with medicines given to treat the disease. This distinction, along with type of crushed root, mode of administration and reason for use should be explored in future studies. Community elders report that numerous traditional medicines are used in the region, made from a variety of roots, stems, bark, and leaves, to treat various maladies including epilepsy (Odonga, unpublished communication).

Contrary to previous findings of a 2001 South Sudan investigation (Anker, unpublished report), our case-control investigation suggests that there is no significant association between NS and a history of measles, a risk factor of interest due to the association of measles with subacute sclerosing panencephalitis, a progressive neurologic complication of an aberrant measles virus infection. In fact, measles by history was less common among cases in the previous study, and slightly more common in ours.

Our investigation adds additional information on the previously identified association between NS and onchocerciasis with the most extensive analyses of the association to date. While other investigations have assessed for an association between onchocerciasis and NS, [7], [9] our investigation provided additional laboratory testing of the cases, and included comparisons with two groups of controls using robust statistical analyses. This association was particularly strong for antibodies measured in the more sensitive OvFAR/MSA LIPS assay, which was positive in 95% of cases. [14] Though the presence of *O. volvulus* microfilariae by skin snip was more common among cases, it was not significantly so. Skin snip testing indicates presence of viable parasites in the skin and may be transient or affected by treatment, while anti-*O. volvulus* IgG antibodies remain present indefinitely, and are a more sensitive indicator for exposure to onchocerciasis.

The association between case status and onchocerciasis is puzzling. Though extensive previous literature debates the relationship between onchocerciasis and epilepsy [15]–[17], the biological mechanism by which *O. volvulus* might cause epilepsy or NS is not clear. *O. volvulus* is typically associated with eye disease by triggering the host immune system leading to ocular tissue destruction. [18] *O. volvulus* is not known to cause invasive infection of the central nervous system. Moreover, there has been no laboratory evidence of the parasite in the brain or CSF as signs of CSF inflammation has been generally absent in NS cases, [7], [10], [16] and Tanzania and South Sudan investigations failed to find evidence of *O. volvulus* by spinal fluid PCR. [6], [7] Also, though all known cases of NS have occurred in onchocerciasis areas, onchocerciasis is endemic in many parts of the world and NS has only been described in limited geographic areas. It is possible that a new antigenic form of *O. volvulus* is present in areas with NS; however, at present there are no data to support this. Additionally, we wondered if children with NS had treatment for onchocerciasis withheld based on the presence of neurologic illness. However no difference was found in reported receipt of treatment between cases and controls (Table 3). Also, it is possible that additional risk factor(s) need to be present to result in NS. Lastly, we cannot determine whether higher levels of anti-onchocerca antibodies present in cases occurred before or after NS onset. In focus groups (UgMOH, WHO, CDC, unpublished report), informants reported that children with NS were less able to swat away black flies given their functionally impaired condition and thus may have been more likely to be bitten by vectors carrying the onchocerca parasite. Alternatively, onchocerciasis may be an indicator of another exposure that is the actual cause of NS.

Cases were malnourished, with 60% having a low height-for-age, and 42% having a low BMI. Exposure questions aimed at assessing nutritional status prior to NS onset, including reported history of malnutrition or consumption of supplementary foods, revealed no difference between cases and controls. In addition, we assessed nutritional biomarker and heavy metal levels, hypothesizing that there may be a role for nutritional deficiencies or intoxications in the development of NS. All cases and controls had low selenium which is likely a reflection of the poor diet quality in this population. Additionally, we found the majority of subjects having low PLP levels. Though both cases and controls have high levels of apparent vitamin B6 deficiency, the findings do raise the question as to whether low vitamin B6 could contribute to disease pathogenesis. Vitamin B6 is important for neurotransmitter synthesis, and depletion can impair neurologic function and result in intractable seizures.[19]–[21] In a controlled depletion-repletion study, [22] 2 of 11 young women placed on a low vitamin B6 diet (<0·05 mg of pyridoxine) exhibited abnormal EEG patterns when plasma PLP levels dropped to about 9 nmol/L. In addition, pyridoxine-dependent seizures, a rare form of early childhood epilepsy, is refractory to usual anti-epileptic medications but can be successfully treated with high doses of pyridoxine. [23].

A number of variables might help explain the unusual nutrition profile with low serum PLP levels, though such an isolated vitamin deficiency is uncommon. Field collection, processing and shipping conditions are unlikely to cause surreptitious low PLP with normal levels of other B vitamin status indicators. A diet limited to un-enriched grains increases the risk for developing pyridoxine deficiency. The Acholi people are agro-pastoralists who relied primarily on relief food while displaced, and were tapered off of relief food as they were resettled. A national food consumption survey in 2008 found that this region of Uganda was unique in inadequate intakes of vitamin B6 containing foods. [24] Because it would be expected that other micronutrients would also be low with low intake of vitamin B6 foods, it is unusual that vitamin B6 is lower than other micronutrients. Certain substances may lower PLP levels, including various drugs (isonazid, cycloserine, penicillamine, hydralazine), smoking, excessive alcohol intake, and consumption of the mushrooms *Agaricus bisporis* and *Gyromitra esculenta*. [25] Additionally, reference values for PLP are not established for this population of children in Sub-Saharan Africa. Finally, it is possible that low levels could be related to a high parasite load. In a model of infection with the filarial parasite *Litomosoides carinii* using rodents, rat microfilariae require vitamin B6 from the host and compete with host cells for vitamin B6; high microfilariae counts correlate with low pyridoxine. [26] However, we were unable to document a significant interaction between onchocerciasis and vitamin B6 deficiency.

A limitation of this investigation is surveillance may under-estimate (e.g. if cases were missed) true prevalence. Also, because the critical period where exposure increases risk of developing nodding is unknown and could be any time from conception to NS onset, our findings may be unable to discern an effect of NS from a potential cause of it. Finally, because we examined multiple associations, some of the associations that we observed may be false positives, however this investigation was intended to be exploratory, with a focus on potential public health interventions, and therefore our findings need to be replicated and expanded in future studies. Despite these limitations, this investigation adds new information to the few small, uncontrolled, or unpublished studies of NS to date.

### Conclusions

We identified *O. volvulus* infection, exposure to munitions, and crushed roots as potential risk factors for nodding syndrome. Consumption of red sorghum, river fish, bush meat, and cassava, drinking water from boreholes, and low selenium levels were found in nearly all cases and controls; low vitamin B6 was also prevalent in most subjects. Future studies should explore additional risk factors within this population and across affected populations, and better evaluate for a combination of exposures as a potential cause. Additionally, expanded surveillance should better describe the prevalence and geographic distribution. While additional investigations are underway to further elucidate the etiology and best treatment regimens, it is important to ensure that mass onchocerciasis treatment campaigns reach populations with high documented rates of infection and that those with known epilepsy and micronutrient deficiencies receive appropriate treatment.

## Supporting Information

Figure S1 Graphical depiction of age distribution: number of nodding syndrome cases indentified by surveillance and case-control studies in Kitgum District by age in years.*
(TIF)

Table S1 Frequency of nodding syndrome cases and village controls with positive exposures. Adjusted odds of positive exposure in a case versus control.
(DOCX)
