# Supplementary figures and images for: An Epidemiologic Investigation of Potential Risk Factors for Nodding Syndrome in Kitgum District, Uganda
Source: PLoS One. 2013 Jun 18;8(6):e66419. doi: 10.1371/journal.pone.0066419 (PMC3688914; doi:10.1371/journal.pone.0066419)

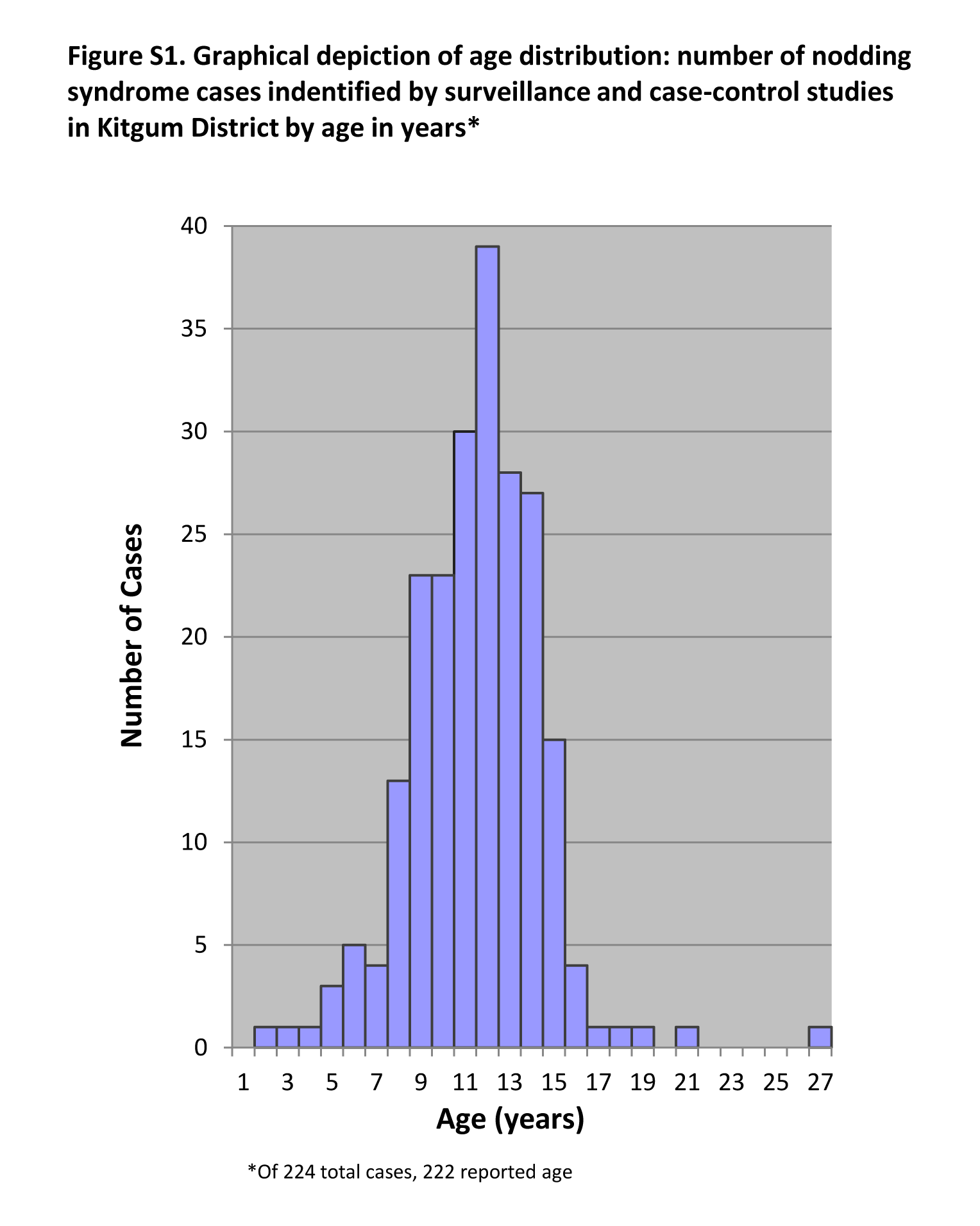

Supplement: Figure S1 — Graphical depiction of age distribution: number of nodding syndrome cases indentified by surveillance and case-control studies in Kitgum District by age in years.* (TIF) [file pone.0066419.s001.tif]
