# Supplementary material for: An Epidemiologic Investigation of Potential Risk Factors for Nodding Syndrome in Kitgum District, Uganda
Source: PLoS One. 2013 Jun 18;8(6):e66419. doi: 10.1371/journal.pone.0066419 (PMC3688914; doi:10.1371/journal.pone.0066419)
Supplement: Table S1 — Frequency of nodding syndrome cases and village controls with positive exposures. Adjusted odds of positive exposure in a case versus control. (DOCX) [file pone.0066419.s002.docx]

| *Table S1*: Frequency of nodding syndrome cases and village controls with positive exposures. Adjusted odds of positive exposure in a case versus control. | | | | | | | | | | | | |  |
| --- | --- | --- | --- | --- | --- | --- | --- | --- | --- | --- | --- | --- | --- |
|  | **Positive Cases**  **%*** | | | | **Positive Controls %** | | | | **AOR Model 2^¶^**  **(95%CI)** | | **AOR Model 3^§^**  **(95%CI)** | |  |
| **Exposure** |  | | | |  | | | |  | |  | |  |
| Ever treated for Onchocerciasis | 33·3 | | | | 24·5 | | | | 1·0 (0·2, 4·7) | | 1·7 (0·4, 7·1) | |  |
| Family member abducted | 58·8 | | | | 49·0 | | | | 0·8 (0·2, 3·6) | | 1·0 (0·3, 3·3) | |  |
| *History of* |  | |  | |  | |  | |  | |  | |  |
| Measles | 23·5 | | | | 6·1 | | | | 1·7 (0·4, 7·9) | | **4·6 (1·0, 21·9)** | |  |
| Malaria^ | 43·1 | | | | 59·2 | | | | 0·9 (0·3, 3·1) | | 0·9 (0·2, 3·1) | |  |
| Malnutrition^ | 3·9 | | | | 4·1 | | | | 10·3 (0·8, 127·8) | | 1·3 (0·2, 7·7) | |  |
| Pneumonia^ | 0·0 | | | | 2·0 | | | | 0·6 (0·0, 37·4)^+^ | | 1·0 (0·0, 66·1)^+^ | |  |
| Diarrhea^ | 2·0 | | | | 4·1 | | | | 2·1 (0·0, 154·6)^+^ | | 1·5 (0·3, 9·1) | |  |
| Head injury^ | 2·0 | | | | 0·0 | | | | 3·9 (0·0, 406·0)^+^ | | 0·3 (0·0, 3·2)^+^ | |  |
| Tapeworm | 0·0 | | | | 0·0 | | | | --- | | --- | |  |
| *Consumption of* |  | |  | |  | |  | |  | |  | |  |
| Red sorghum | 98·0 | | | | 100 | | | | 1·8 (0·0, 184·8)^+^ | | 1·4 (0·0, 149·1)^+^ | |  |
| Spoiled relief foods | 243·1 | | | | 46·9 | | | | 0·5 (0·1, 2·5) | | 0·6 (0·1, 2·8) | |  |
| Supplementary foods | 21·6 | | | | 12·2 | | | | 0·9 (0·2, 4·8) | | 2·2 (0·5, 9·5) | |  |
| Seeds meant for planting | 60·8 | | | | 65·3 | | | | 0·3 (0·1, 1·5) | | 0·6 (0·1, 2·7) | |  |
| River Fish | 96·1 | | | | 100 | | | | 1·8 (0·0, 124·7)^+^ | | 0·1 (0·0, 15·0)^+^ | |  |
| Insects | 41·2 | | | | 32·7 | | | | 1·1 (0·2, 5·8) | | 0·6 (0·1, 2·5) | |  |
| Rodent brain | 54·9 | | | | 51·0 | | | | 1·7 (0·2, 12·3) | | 3·7 (0·4, 33·4) | |  |
| Guinea fowl brain | 7·8 | | | | 4·1 | | | | 0·9 (0·1, 8·3) | | 7·5 (0·5, 104·1) | |  |
| Bush meat | 100 | | | | 100 | | | | --- | | --- | |  |
| Cassava | 100 | | | | 100 | | | | --- | | --- | |  |
| *Use of traditional or herbal medicines* | | | | | | | | | | | | |  |
| Crushed roots^ | | 39·2 | | | | 16·3 | | | | 4·4 (0·9, 21·1) | | **6·7 (1·3, 33·1)** | |
| Crushed leaves^ | | 7·8 | | | | 2·0 | | | | 3·8 (0·1, 128·8) | | 4·1 (0·3, 59·0) | |
| Crushed flowers^ | | 0·0 | | | | 2·0 | | | | --- | | 1·2 (0·1, 10·0) | |
| Inhaled medicine^ | | 2·0 | | | | 0·0 | | | | 0·3 (0·0, 28·2)^+^ | | 0·2 (0·0, 2·4)^+^ | |
| Lotion | | 0·0 | | | | 0·0 | | | | --- | | --- | |
| Broth | | 0·0 | | | | 0·0 | | | | --- | | --- | |
| *Water source for domestic use* | |  | |  | |  | |  | |  | |  | |
| Rivers/streams | | 80·7 | | | | 83·7 | | | | 0·2 (0·0, 7·8)^+^ | | 0·0 (0·0, 1·9)^+^ | |
| Boreholes | | 96·1 | | | | 100 | | | | 0·3 (0·0, 17·6)^+^ | | 0·1 (0·0, 6·6)^+^ | |
| Shallow wells | | 11·8 | | | | 6·1 | | | | **6·4 (2·0, 20·6)** | | 6·9 (0·4, 108·8)^+^ | |
| Springs | | 5·9 | | | | 4·1 | | | | 2·8 (0·3, 25·7) | | 1·9 (0·1, 34·3) | |
| Piped water | | 0·0 | | | | 0·0 | | | | --- | | --- | |
| *Exposure to* | |  | |  | |  | |  | |  | |  | |
| Munitions | | 70·6 | | | | 51·0 | | | | **10·4 (1·0, 107·7)** | | **17·2 (1·3, 226·4)** | |
| Unusual illness/death of animals | | 52·9 | | | | 53·1 | | | | 1·4 (0·3, 7·0) | | 0·3 (0·0, 1·7) | |
| Swimming in the river^ | | 17·7 | | | | 22·5 | | | | 0·2 (0·0, 1·8) | | 0·4 (0·1, 2·6) | |
| Swimming in the pond^ | | 7·8 | | | | 4·1 | | | | 1·9 (0·1, 42·2) | | 2·6 (0·0, 311·0) | |
| *Statistically significant values are in bold.*  *CI: Confidence interval. OR: odds ratio.* | | | | | | | | | | | | | |
| ** Percent with exposure is calculated by number of cases with a positive exposure divided by number of cases, or number of controls exposed divided by number of controls* | | | | | | | | | | | | | |
| *‡ Odds ratio calculated as odds of positive exposure in cases versus odds of exposure in controls* | | | | | | | | | | | | | |
| **¶***AOR_2_: Odds ratio adjusted for age, munitions, roots* | | | | | | | | | | | | | |
| **§***AOR_3_: Odds ratio adjusted for age, measles, sorghum, onchocerciasis skin snip positive* | | | | | | | | | | | | | |
| *^Missing data existed for the following exposure variables: malaria, malnutrition, pneumonia, diarrhea, head injury, crushed leaves, roots, flowers, inhaled medicine (number of cases responding to question=50); swimming in the river or pond, (cases=49); all data used for frequencies, data from available matched pairs used for matched analyses.* | | | | | | | | | | | | | |
| *+Firth’s correction* | | | | | | | | | | | | | |
